# Long-Term Follow-Up of Survivors of Extracorporeal Life Support Therapy for Cardiogenic Shock: Are They Really Survivors?

**DOI:** 10.3390/medicina58030427

**Published:** 2022-03-15

**Authors:** Rafal Berger, Attila Nemeth, Rodrigo Sandoval Boburg, Louise Vöhringer, Henning Florian Lausberg, Metesh Acharya, Christian Schlensak, Aron-Frederik Popov

**Affiliations:** 1 Department of Thoracic and Cardiovascular Surgery, University Hospital Tübingen, 72076 Tübingen, Germany; attila.nemeth@med.uni-tuebingen.de (A.N.); rodrigo.sandoval-boburg@med.uni-tuebingen.de (R.S.B.); louise.voehringer@med.uni-tuebingen.de (L.V.); christian.schlensak@med.uni-tuebingen.de (C.S.); aronf.popov@gmail.com (A.-F.P.); 2 Department of Thoracic and Cardiovascular Surgery, Saarland University Medical Center, 66421 Homburg, Germany; lausbergh@me.com; 3 Department of Cardiac Surgery, Glenfield Hospital, Leicester LE3 9QP, UK; metesh.acharya@doctors.org.uk; 4 Department of Cardiac Surgery, Helios Heart Center Siegburg, 53721 Siegburg, Germany

**Keywords:** cardiogenic shock, extracorporeal membrane oxygenation, long-term follow-up

## Abstract

*Background and Objectives:* Cardiogenic shock (CS) is a medical emergency associated with a high mortality rate. Veno-arterial extracorporeal membrane oxygenation (VA-ECMO) has become an accepted therapy for CS. Despite widely available data for short-term survival rates, there are only limited data available regarding long-term outcomes following successful VA-ECMO therapy. *Materials and Methods:* We analyzed the demographics, past medical history, adverse events, and outcomes of survivors who received VA-ECMO support for CS at our center from January 2012 to December 2019. Post-cardiotomy cases were excluded. *Results:* A total of 578 VA-ECMO implantations on 564 consecutive patients due to CS were identified during the study period. Successful weaning was achieved in 207 (36.7%) patients. Among the survivors, 126 (63%) patients received VA-ECMO therapy without preceding cardiac surgery during their current admission. A follow-up exceeding 12 (mean: 36 ± 20.9) months was available in a total of 55 (43.7%) survivors. The mean VA-ECMO perfusion time was 10.9 (±7.7) days with a mean intensive care unit (ICU) stay of 38.2 (±29.9) days and a mean hospital stay of 49.9 (±30.5) days. A total of 3 deaths were recorded during long-term follow-up (mean survival of 26 ± 5.3 months). *Conclusions:* Despite the high mortality associated with VA-ECMO therapy, a long-term follow-up with an acceptably low rate of negative cardiac events can be achieved in many survivors. We observed an acceptable low rate of new cardiac events. Further evaluation, including a quality-of-life assessment and a close follow-up for rarer complications in these patients, is needed to elucidate the longer-term outcomes for survivors of invasive VA-ECMO therapy.

## 1. Introduction

Cardiogenic shock (CS) is a medical emergency characterized by reduced cardiac output with organ hypoperfusion. The associated 40–90% mortality rate depends on the specific underlying etiology, patient condition, and applied therapy [1,2]. Only the rapid identification of clinical and biochemical manifestations of inadequate tissue perfusion, followed by effective treatment of CS, improves the outcome [3]. Veno-arterial extracorporeal membrane oxygenation (VA-ECMO) has become a mainstream accepted therapy for CS in patients who have failed to respond to conventional medical treatment such as intravascular volume replacement, inotropic pharmacotherapy, or other forms of mechanical circulatory support (MCS) [1,3,4,5].

The goal of effective VA-ECMO therapy is to maintain sufficient and continuous organ perfusion until cardiac function recovers (bridge-to-recovery) or to facilitate subsequent intervention—for example, cardiac transplantation (bridge-to-transplantation) or implantation of a left ventricular assist device (LVAD, bridge-to-bridge). Successful acute treatment stabilizes the patient and affords more time for ongoing clinical decision making (bridge-to-decision) [6].

Nevertheless, rescue therapy with VA-ECMO is associated with numerous complications such as bleeding, stroke, renal or liver failure, lower-extremity ischemia, infection, and thrombosis [6,7]. These adverse effects often result in mortality or permanent injury [7]. The Extracorporeal Life Support Organization’s (ELSO) registry reports successful weaning of 56% and survival-to-discharge in 42% of adult patients receiving VA-ECMO for CS in 2016 [7]. The technique also mandates considerable financial and human resources to support often-extended durations of therapy, averaging 144 h in the latest summary by ELSO [6,7].

Despite widely available results for short-term survival rates, only limited data are presented for longer-term outcomes after successful support with VA-ECMO [3,6,8]. The fate of survivors remains unknown after discharge. Our study, therefore, sought to analyze the indications, pre-implantation status, and outcome of non-cardiac surgical patients who survived VA-ECMO therapy. The purpose of our investigation was to evaluate the duration of survival and to assess the complications after discharge from the clinic and to answer the question of whether an event-free follow-up is possible in patients successfully therapied with VA-ECMO.

## 2. Materials and Methods

### 2.1. Patient Selection

We performed a retrospective analysis of all consecutive adult patients who received extracorporeal life support (ECMO) therapy for CS in our center or by our mobile team between January 2012 and December 2019. A follow-up was completed in April 2020 and was based on voluntary outpatient control offered to every survivor after discharge. Post-cardiotomy cases were excluded from further analysis. To better analyze the long-term follow-up of survivors, only patients who survived for more than a year after initial treatment were further analyzed.

The primary end-point was survival backed up with a completed follow-up. The secondary end-point was the appearance of complications requiring further therapy in VA-ECMO survivors.

### 2.2. Statistical Analysis

Statistical analysis was performed by using SPSS 26 (IBM Corporation, Armonk, NY, USA) software. Continuous variables are reported as means and standard deviations for normal distributions and as median ranges for non-normal distributions. The Kolmogorov–Smirnov test was performed to check for the distribution of these variables. Ordinal variables are reported as absolute values and percentages where applicable. Variables were compared by using Fisher’s exact or Mann-Whitney U tests where applicable.

### 2.3. Ethical Approval

Ethical approval for this study was sought from the Ethics Committee Board at our institution (Ref. 194/2020BO2 from 15 April 2020). Due to the retrospective nature of the study, the need for written consent was waived.

## 3. Results

Over the study period January 2012–December 2019, a total of 578 VA-ECMO implantations were performed on 564 consecutive patients. There were 11 early reimplantations for patients during their current admission and 3 late reimplantations following hospital discharge. A total of 212 (36.7%) VA-ECMO runs resulted in successful device weaning in 207 (36.7%) patients (including 5 (2.4%) patients after 2 VA-ECMO implantations). Weaning and living explantation were counted if the patient survived at least 24 h without ECLS. There were seven deaths in the 24-h period following successful explantation, resulting in 200 (35.5%) patients being alive at discharge.

**Figure 1:**
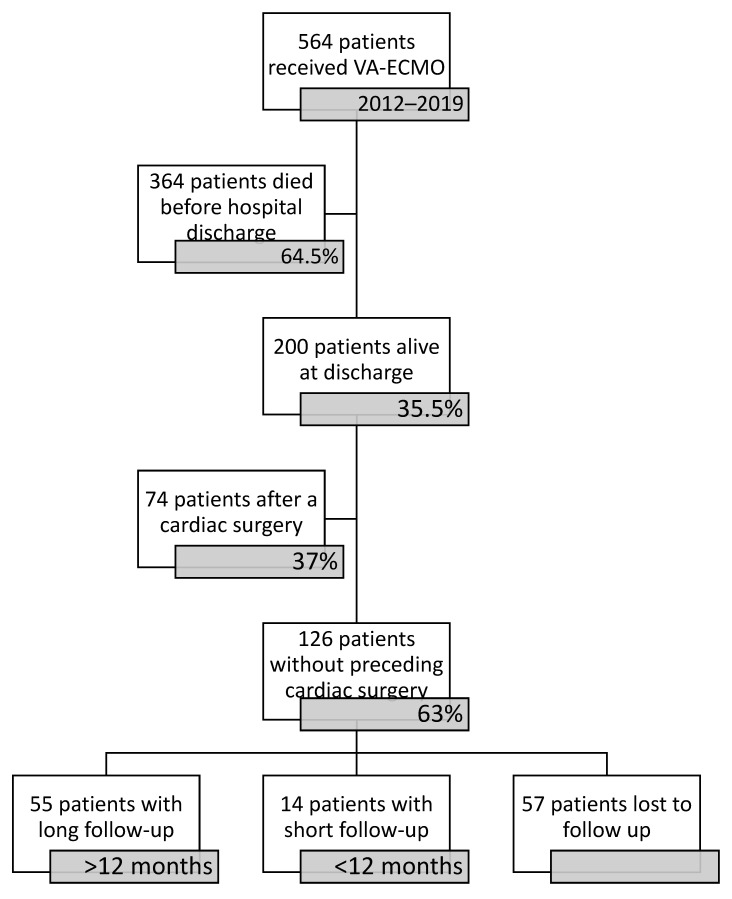
Figure of participants.

In surviving patients, there were 74 (37%) cases of MCS subsequent to cardiac surgery performed on the current admission. These patients were excluded from further analysis. From a total of 126 VA-ECMO survivors, 34 (27%) patients were lost to follow-up directly after discharge. In 14 (11.1%) cases, follow-up was shorter than 12 months and in 23 (18.3%) cases, follow-up data were not available beyond 12 months (Figure 1). There was only one case of more than one VA-ECMO run in the group of survivors. In a total of 55 patients (43.7% of ECLS therapy due to CS), a follow-up of over 12 months could be completed. Patient characteristics are listed in Table 1.

The mean time of VA-ECMO perfusion was 10.9 ± 7.7 days. While on support, bleeding requiring intervention (surgical or percutaneous) was the most common complication and occurred in 21 (38.2%) cases. The implantation of LVAD was by far the most common therapy in patients who were unable to wean from VA-ECMO, and it was performed in 26 (47.3%) patients. Table 2 shows the complications encountered by the study population while on VA-ECMO and their associated management.

Complications arising during the follow-up period are summarized in Table 3. The mean duration of follow-up was 36 ± 20.9 months. A total of three deaths were recorded with a mean survival of 26 ± 5.3 months. In four (15.4%) patients receiving an LVAD, a successful explantation was performed after a mean support time of 18.6 ± 11.5 months and included two cases of myocarditis, one case of arrhythmogenic cardiomyopathy, and one case of post-partum cardiomyopathy. Renal replacement therapy (*n* = 11, 20%), wound infections (*n* = 10, 18.2%), and cerebrovascular accidents (*n* = 6, 10.9%) were the most common complications occurring during follow-up.

**Figure 2:**
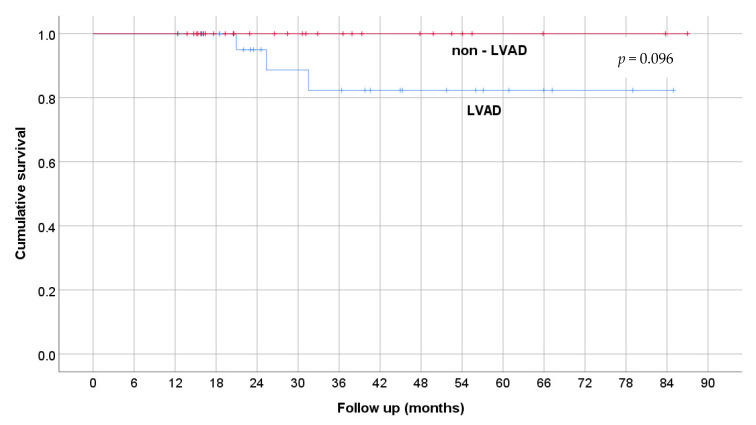
Kaplan–Maier curve of survival.

The outcomes of survivors were compared between LVAD recipients and patients discharged without MCS, as shown in Table 4. The duration of VA-ECMO support, hospital stay, and length of follow-up wasnot significantly different between groups. According to the characteristic data shown in Table 4, only exacerbations of chronic heart failure occurred significantly (*p* = 0.001) and more often in the subpopulation bridged with LVAD. The Kaplan–Maier survival curve of patients who had left the hospital alive is shown in Figure 2.

## 4. Discussion

ECLS therapy has demonstrated encouraging in-hospital survival rates, but the longer-term outcomes have not yet been fully ascertained [9]. Blumenstein et al., compared a group of patients who underwent ECLS implantation under cardiopulmonary resuscitation (ECPR) with a propensity score-adjusted group who received conventional cardiopulmonary resuscitation (CPR) [9]. A significant difference in long-term survival (23.1% vs. 11.5%) favored ECPR during the median follow-up duration of 1136 days after discharge [9]. Our study presents the longer-term outcomes of VA-ECMO survivors over an 8-year period at a large tertiary care center. With mounting supporting evidence, VA-ECMO therapy has been demonstrated as an effective method for hemodynamic support in low cardiac-output states [1]. Nevertheless, mortality remains high. According to ELSO’s data registry, CS was the most common cardiac indication in adult patients with over 2000 runs and with successful ECMO explantation in 56% of cases and an overall 42% survival-to-discharge in 2016 in participating centers [7]. The outcomes were poorer in the ECPR group of patients with 39% of patients weaned and 29% discharged after ECMO therapy [7]. The outcomes of ECMO therapy in our study population are similar, resulting in 35.5% survival-to-discharge, and are the basis for further analysis of long-term survivors. El Sibai et al. analyzed public US Nationwide Emergency Department Sample (NEDS) data for 2013. They noted 8,605,807 adult emergency department visits for a diagnosis of CS, in whom 992 consecutive ECLS procedures were performed, yielding an MCS rate of 0.1 per 1000 admissions [8]. Muller et al. analyzed the outcomes of 138 patients treated with ECLS for CS related to acute myocardial infarction. After initial survival-to-discharge in 47% of cases, a follow-up and quality of life could be assessed in 41 of 57 (77%) long-term survivors [10]. However, the observational period was only 12 months, which does not reflect a real-world scenario. In our patient population, successful weaning was observed in 36.7% of patients and survival-to-discharge was observed in 35.5%. We were able to achieve a longer observation period due to our close collaboration within the heart failure team, and so we could present a mean follow-up of 36 months after ECLS therapy.

There are currently no defined or evidence-based criteria for the initiation of VA-ECMO in patients with CS [11]. At our center, implantation is considered in cases of CS with progressive tissue hypoperfusion refractory to medical therapy, when the condition leading to CS is presumed to be reversible or eligible for further therapy. There are no strict cut-off criteria, but pH, lactate level, and biomarkers of organ damage are important parameters that should be carefully taken into account. Exclusion criteria are also not strictly defined, but a short life expectancy due to other medical conditions, cerebral hemorrhage, aortic dissection, severe trauma, advanced age, or previous ”do not resuscitate” orders are considered [2,9]. The final decision is always made by the cardiothoracic surgeon in consensus with the intensivist. At our institution, weaning from VA-ECMO is performed according to departmental protocols concomitant to an improvement of cardiac function, failure of further bridging with LVAD, or for evaluation for cardiac transplantation. Severe complications or lack of further possible therapy after failed weaning are followed by a the withdrawal of further life support. In our group, one patient deteriorated after successful weaning of VA-ECMO and required a second device run, which resulted in LVAD implantation shortly thereafter.

Due to the retrospective character of the study, we decided to exclude the patients in CS due to previously performed cardiac surgery. We made this decision because this group includes VA-ECMO runs due to a critical perioperative state and complex procedure and also involved MCS because of perioperative complications of the surgery itself.

As cardiac transplantation is not available in our institution, successful weaning or bridging with an LVAD are the only two possible outcomes in survivors of VA-ECMO therapy. The pre-ECLS cardiac arrest and the acute exacerbation of a chronic cardiac disease were the only significant differences between survivors of LVAD and non-LVAD groups (Table 4). This may raise concerns that cardiac events such as cardiogenic shock due to acute infarction or a malignant arrhythmia in patients without a previous chronic heart failure diagnosis may primarily manifest as cardiac arrest but are more prone to successful weaning and, despite the implantation of LVAD as a ”bridge-to-bridge” therapy, a long-term follow-up can be achieved. In another publication of the authors, the quality of life after LVAD implantation while on VA-ECMO was shown to be non-inferior when compared to elective LVAD recipients [12]. This, in our opinion, makes the implantation of LVAD a first-line therapy in patients unable to wean from VA-ECMO.

Schmidt et al. proposed the Survival After Veno-arterial-ECMO (SAVE) score to predict in-hospital mortality after ECLS use in CS [6]. The authors found that the initial indication for ECLS played a significant role in outcomes, and more reversible causes such as myocarditis or arrhythmias were associated with enhanced short-term outcomes [6]. Furthermore, younger age, shorter intensive care unit (ICU) stays prior to ECLS institution, and the lack of central nervous system dysfunction or liver or renal failure were associated with enhanced survival [6]. These findings were corroborated by Truby et al. who determined that younger age and etiology are the most influential factors affecting short-term outcomes [13].

The mean age of the group of survivors at the time of VA-ECMO initiation was 58 +/− 12.9 years, and mean survival reached 36 +/− 20.9 months. In 26 (47.3%) patients, bridging with LVAD was required for further support with consideration toward possible explantation, as performed in four (15.4%) cases. We additionally employed other modalities of temporary MCS with good results. As depicted in Table 2, potential RVAD implantation for right heart failure (five patients, 9.1%), conversion to veno-venous (VV) ECLS (two patients, 3.6%), and the step-wise reduction in invasiveness with an intra-aortic balloon pump (IABP, four patients, 7.3%) or micro-axial pump (Impella^®^, Abbott, Abbott Park, IL, USA; seven patients, 12.7%) support representative adjunctive therapies, which can extend survival.

The fate of VA-ECMO survivors remains largely unknown and has not been thoroughly investigated. The majority of studies assessing this phenomenon concentrate on treatment outcomes and survival-to-hospital discharge. Only a handful of studies focus on long-term survival beyond 1 year after weaning off ECLS [9]. Burrell et al. determined that good long-term survival could be achieved following ECLS, observing 79% survival at 12 months [14]. However, follow-up in their study was increasingly incomplete for time intervals exceeding 12 months, with survival data available for only 66% of patients at 24 months [14]. Ørbo et al. evaluated heart-related quality of life after ECLS [15]. They identified 30 (41%) of 74 ECLS-survivors in Norway and surveyed 23 survivors, with 40% of respondents reporting some degree of restriction in everyday activities and depression in 35% of cases [15]. Camboni et al. assessed the quality of life after a mean follow-up period of 1598 days in 82 (44.8%) of 183 ECLS survivors [16]. In our series, we focused on survival and medical complications after initial successful VA-ECMO therapy. In 126 identified cases of survival due to CS, only 55 (43.7%) patients had a follow-up period of greater than 12 months, mostly due to a lack of standard aftercare. Nevertheless, as reported in Table 3, event-free, long-term survival can be achieved. We observed that rates of further cardiac intervention in our cohort remain acceptably low with a cumulative 15 (27.3%) total cardiac interventions during 87 months of follow-up.

In the group of LVAD recipients, explantation was successful in four cases with a mean support time of 18.6 months. The decision to wean the device was made individually after recovery of cardiac function.

Several studies have reported that ECLS implantation can increase the risk of death and identified ECLS initiation as a risk factor associated with in-hospital mortality [9,17]. ECLS-related complications, such as bleeding and limb ischemia, influence both the outcome of the therapy and the subsequent quality of life in survivors. In our study population, 11 (20%) patients required renal replacement therapy during follow-up. Chronic wound issues necessitating surgical revision developed in 10 (18.2%) survivors. Neurological disorders were observed in six (10.9%) long-term survivors. There were no incidences of new CS requiring ECLS during follow-up.

### Limitations

Our study is limited to experience from a single center. The indications and management of VA-ECMO are largely center-specific and variations in treatment, with respect to anticoagulation, mobilization, or terminal weaning, for example, may significantly impact outcomes. Due to the retrospective nature of our study, data on the variables collected may not accurately correspond with patients’ actual medical status. The heterogeneity of the study population has an additional impact on the outcomes, as survival with an LVAD device brings further device-related complications that are not observed in survivors without the assist device. The provision of ECLS support is resource-intensive and may present financial and logistical difficulties for smaller centers. Finally, our patient population was unselected, heterogeneous, and limited to the surgical intensive care ward.

## 5. Conclusions

Despite being a retrospective analysis, we present satisfactory long-term outcomes in survivors of VA-ECMO therapy for CS over an 8-year period. The rate of new cardiac events requiring therapy in our study population was acceptably low. Further prospective analyses incorporating close follow-up and quality-of-life assessments for survivors of ECLS are necessary. The correct application of VA-ECMO therapy for CS is challenging, but despite common complications, long-term results can be achieved and should be followed.

## Figures and Tables

**Table 1:** Patient characteristics, *n* = 55.

Age (Years) Male	58.2 ± 12.9 42 (76.4)
Diagnoses associated with cardiogenic shock:	
Acute myocardial infarction	31 (56.4)
Myocarditis	4 (7.3)
Pulmonary embolism	2 (3.6)
Refractory ventricular VT/VF	19 (34.5)
Sepsis	2 (3.6)
Chronic heart failure	22 (40)
Complication of cardiac intervention	2 (3.6)
Other indications	2 (3.6)
Myocardial function before implantation:	
Left heart failure	35 (63.6)
Right heart failure	13 (23.6)
LV ejection fraction (%)	17.7 ± 10.1
Pre-ECLS cardiac arrest	34 (61.8)
CPR time (min)	28.5 ± 22.8
Implantation under CPR	8 (14.4)
Pre-ECLS IABP/Impella^®^	2 (3.6)
Implantation out of centre	13 (23.6)
Duration of support (days)	10.9 ± 7.7
ICU stay (days)	38.2 ± 29.9
Hospital stay (days)	49.9 ± 30.5

Data are given as n (%), mean ± standard deviation. CPR, cardiopulmonary resuscitation; ECLS, extracorporeal life support; IABP, intra-aortic balloon pump; ICU, intensive care unit; LV, left ventricle; VF, ventricular fibrillation; VT, ventricular tachycardia.

**Table 2:** Complications during VA-ECMO support and their management, *n* = 55.

Complications while on support:	
Bleeding requiring invasive intervention	21 (38.2)
NOMI	3 (5.5)
Stroke	2 (3.6)
LV Thrombus	2 (3.6)
Limb ischaemia	1 (1.8)
Management:	
LVAD-implantation	26 (47.3)
Vascular surgery to treat complications	17 (30.9)
PCI	11 (20)
Other cardiac surgery	7 (12.7)
Impella^®^	7 (12.7)
Temporary RVAD	5 (9.1)
Pacemaker	4 (7.3)
IABP	4 (7.3)
Catheter ablation	2 (3.6)
VV-ECMO	2 (3.6)
Re-VA-ECMO	1 (1.8)

Data are presented as n (%). ECMO, extracorporeal membrane oxygenation; ECLS, extracorporeal life support; IABP, intra-aortic balloon pump; LV, left ventricle; LVAD, left ventricular assist device; NOMI, non-occlusive mesenteric ischaemia; PCI, percutaneous coronary intervention; VA, veno-arterial; VV, veno-venous; RVAD, right ventricular assist device; Impella (Abiomed, Danvers, MA, USA)

**Table 3:** Complications during survivor follow-up, *n* = 55.

Follow-up duration (months)	36 ± 20.9
Deaths	3 (5.5)
Survival to death (months)	26 ± 5.3
Renal failure	11 (20)
Wound infection	10 (18.2)
Cerebrovascular accident	6 (10.9)
New episode of cardiogenic shock	1 (1.8)
Myocardial infarction	1 (1.8)
Pulmonary embolism	1 (1.8)
Hypoxic neurologic injury	1 (1.8)
Depression	1 (1.8)
Implantation of pacemaker	4 (7.3)
PCI	2 (3.6)
MitraClip^®^	2 (3.6)
Catheter ablation	1 (1.8)
Heart transplantation	1 (1.8)
Other cardiac surgery	1 (1.8)
LVAD-explantation	4 (7.3)
Time on LVAD (months)	18.6 ± 11.5

Data are presented as n (%), mean ± standard deviation. LVAD, left ventricular assist device; PCI, percutaneous coronary intervention; MitraClip (Abbott, Menlo Park, CA, USA).

**Table 4:** Comparison between LVAD and non-LVAD recipients.

	LVAD, *n* = 26	Non-LVAD, *n* = 29	*p* Value
Diagnosis:			
Acute myocardial infarction	14	17	0.372
Myocarditis	3	1	0.253
Refractory ventricular VT/VF	8	11	0.581
Chronic heart failure	18	4	0.001 *
CPR:			
Pre-ECMO cardiac arrest	10 (38.5)	16 (55.2)	0.01 *
CPR time (min)	10 (1–60)	25 (1–75)	0.122
Follow up after VA-ECMO implantation:			
Time on VA-ECMO (days)	13 ± 8.1	9.7 ± 6.3	0.096
ICU stay (days)	42.7 (28.4)	38.25 (29.9)	0.523
Hospital stay (days)	60.4 ± 38.5	45.24 ± 29.3	0.105
Follow-up (months)	38.4 ± 21.2	33.8 ± 20.7	0.281

Data are presented as n (%), mean ± standard deviation or median (range). Multiple diagnoses per patient are possible. An asterix (*) marks a statistically significant difference between the two groups; CPR, cardiopulmomnary resuscitation; ECLS, extracorporeal life support; ICU, intensive care unit; LVAD, left ventricular assist device.

## Data Availability

Not applicable.
